# An elusive debate on the evidence for RNA editing in SARS-CoV-2

**DOI:** 10.1080/15476286.2024.2321032

**Published:** 2024-03-01

**Authors:** Giorgio Mattiuz, Salvatore Di Giorgio, Silvestro G. Conticello

**Affiliations:** a Department of Experimental and Clinical Medicine, University of Florence, Firenze, Italy; b German Cancer Research Center (DKFZ) - Division of Immune Diversity, Foundation under Public Law, Heidelberg, Germany; c Core Research Laboratory, ISPRO, Firenze, Italy; d Institute of Clinical Physiology, National Research Council, Pisa, Italy

Dear Editor,

In a recent review [1] Pu and Colleagues discuss a supposedly ongoing debate on the source of mutations in the SARS-CoV-2 virus: many have reported a bias in intra- and inter-host viral mutations, leading to the hypothesis that host deaminases act on the virus [e.g [2–25], and a few noted that – especially for A>G changes – specific pipelines should be used to avoid overestimates [7,26,27].

Our hypothesis stemmed from the striking difference between SARS-CoV replication errors shown in Smith et al. [28] (with an enrichment in transversions over transitions, and T>G being the main nucleotide change) and the profile we, and others, have observed. The lack of enrichment for C>U and A>G misincorporation by the viral replication complex has been, indeed, confirmed recently in vitro [29].

The Authors of the review criticize our original analysis [2] based on the poor separation between ‘editing’ signal from other mutational signals, and on the symmetry of the mutational profiles (C>T levels similar to G>A ones; A>G levels similar to T>C ones). The Authors suggest that this presumed flaw points to either replication or sequencing errors. While we agree that intra-host datasets may be biased by sequencing errors, a similar profile is observed also in inter-host datasets (which derive from consensus sequences, typically unaffected by background noise due to sequencing errors).

The authors support their suggestion on the basis of having redrawn in Fig. 1A our own data. In fact, the graph in this figure looks quite different from our own presentation of the data, both when intra-host or inter-host data are considered [2]. We have tried to obtain their figure using our data, but despite our efforts we have been unable to obtain the pattern (Fig. 1A) that is central to their argument. We thus stand by our original hypothesis and, for a longer discussion on the ‘debate’, we refer to a past commentary [30].

In the end, it might be useful to remember that final evidence for the role of the host deaminases in the evolution of the SARS-CoV-2 will only be experimental. While nothing is available yet for the ADARs, initial experimental evidence on the role of the APOBECs is already available [31,32], which seems to have escaped the Authors’ attention.
